# Let’s not take DNA breaks for granted. The importance of direct detection of DNA breaks for the successful development of DDR inhibitors

**DOI:** 10.3389/fcell.2023.1118716

**Published:** 2023-03-09

**Authors:** Kamil Solarczyk, Magdalena Kordon-Kiszala

**Affiliations:** intoDNA S.A, Kraków, Poland

**Keywords:** STRIDE, DNA damage, DNA damage response (DDR), PD biomarker, DNA breaks, γH2AX, RAD51, comet assay

## Abstract

Successful development of a drug candidate requires availability of robust methods that enable precise and quantitative assessment of the biological effects exerted by the molecule of interest. In case of DNA Damage Response inhibitors, the most proximal readout of their efficiency is the level of induced DNA damage, usually - DNA breaks. Here we review the methods that are currently used for the assessment of the level of DNA damage, with special attention to their specificity and sensitivity. We also discuss the most common problems and challenges related to the classic IF or IHC methods that indirectly report on the activation of DNA repair mechanisms as the downstream effects of occurrence of the DNA lesions. Finally, we highlight the advent of new tools, such as STRIDE, which have the potential to transform the landscape of DDR functional biomarkers.

## Introduction

Cancer therapy based on inhibition of DNA repair pathways still seems a relatively new concept (Farmer et al., 2005), yet cancer treatment and DNA damage have been fundamentally intertwined since the very beginning of chemotherapy. The simple, but unfortunately, not always achievable goal of these pioneering approaches was to kill proliferating cancer cells more efficiently than their non-malignant counterparts. What was not known for the researchers at the time of conception of those therapeutic interventions was that most of the compounds used, indirectly or directly interfere with the DNA and in turn lead to formation of DNA damage (Markovits et al., 1987). Years of research have deepened our understanding of the mechanisms of action of chemotherapeutics, showing that they may lead to a variety of DNA lesions, from alkylations, oxidations, and crosslinks, through DNA nicks and ssDNA gaps to the most deleterious of all—double-strand DNA breaks (Tilsed et al., 2022). That knowledge currently helps to design better combinations and schedules of treatment. Although unintentionally at first, the weakness of cancer cells, i.e., their suboptimal capability to repair DNA damage, was turned into the strength of our therapeutic strategies. Uncovering the genetic makeup of cancer cells have pushed our hopes even further, leading to the conception of synthetic lethality within DNA Damage Response (DDR) and then to the approval of first PARP inhibitors (Bryant et al., 2005; Fong et al., 2009; Geenen et al., 2018; Golan et al., 2019). Currently, the DDR drug discovery field seems to be thriving with drug candidates in clinical trials and lots of new targets in early development (Cheng et al., 2022). The success of PARP inhibitors would not have been possible without a careful choice of PD biomarkers, in this case—the level of poly (ADP-ribose) (PAR) polymers. The decrease in the level of PARylation elegantly informed about the effect of the drug. However, it is not always straightforward to find a suitable biomarker, especially when the precise mechanism of action of the drug remains unknown or in the case of combination treatments. It is thus crucial to realize that while novel DDR inhibitors target proteins from different pathways and their mechanisms of action vary, the ultimate goal for these strategies is the same—to kill a cancer cell by increasing the level of DNA damage, usually DNA breaks, to a level which exceeds the capacity of DNA repair mechanisms. Thus, DNA breaks level can be thought of as a unifying and proximal readout (PD biomarker) across multiple DDR inhibitors and, as a consequence, the methods that are used for measuring the level of DNA integrity are of great importance for the development of this class of drugs. In this mini review we summarize methods that can be used to assess the level of DNA damage—both the classic approaches, such as immunolabeling of repair proteins or the comet assay and relatively new techniques, like DNA breaks profiling or STRIDE. We discuss their general strengths and weaknesses as well as the perspective on their applicability and usefulness in different phases of drug development.

## IF- and IHC-based microscopy detection of the DNA Damage Response

The enormous leap in our understanding of the DDR in the last 20 years would not have been possible without the development of immunofluorescence (IF)- and immunohistochemistry (IHC)-based microscopy methods aimed at detecting players engaged in various DDR pathways. A classic example of such methods is the detection of γH2AX—a phosphorylated form of H2AX histone variant (Rogakou et al., 1998). Phosphorylation of H2AX happens relatively quickly after formation of a double-strand DNA break (DSB) and occurs in the vicinity of the lesion. After its discovery, this biochemical modification soon became the gold standard in detection and quantification of DNA damage. The unprecedented success of the method is probably due to a couple of factors, among which the most important are straightforward and non-laborious protocol, relatively high signal-to-noise ratio in the obtained images resulting from the naturally occurring biological amplification and quick generation of quantitative data. Since its introduction, the γH2AX assay has been used in a plethora of experiments, some of which were fundamental to our current understanding of the DNA repair processes and cancer in general (Lee and Paull, 2004; Bartkova et al., 2005; Bryant et al., 2005; Gorgoulis et al., 2005). Over the years, owing to its widespread use, detecting γH2AX became almost equal to the presence of DNA breaks. However, there seems to be a growing body of evidence that although usually related to a stress reaction, γH2AX foci formation might also occur in the absence of physical DNA breaks, which puts their usefulness for DNA damage level measurements into question. First, it was shown that UV irradiation leads to formation of γH2AX foci which are often not associated with 53BP1 foci, another marker of DSBs, or results in pan-nuclear γH2AX signals, probably a pre-apoptotic marker (de Feraudy et al., 2010). Subsequent studies have confirmed that γH2AX foci in UV-treated S-phase cells are probably replication-stress related and are not associated with primary (cyclobutane pyrimidine dimers, CPDs) nor secondary damage (DSBs) supposed to be formed after such insult to cellular DNA (Dhuppar et al., 2020). Importantly, the appearance of γH2AX foci not related to DNA breaks has been extended beyond UV irradiation. Various chemotherapeutics, including topoisomerase inhibitors, have been shown to produce γH2AX foci which are unrelated to DSBs or even to DNA replication, hinting at the possibility of γH2AX marking other DNA lesions apart from DSBs or being a more general indicator of distortion of DNA structure (Rybak et al., 2016). The latter seems to be especially important when considering inhibitors of DNA repair proteins such as PARP1, POLQ or RAD51 which act on the DNA molecule (Scott et al., 2021; Stockley et al., 2022; J; Zhu et al., 2015). The interpretation of γH2AX signals is further complicated by the presence of the already mentioned and usually transient pan-nuclear signals (Meyer et al., 2013), which can be triggered not only by UV but also with hypotonic treatment or viral infection (Cuconati et al., 2003; Blackford et al., 2008; Nichols et al., 2009). Also, localized ionizing radiation may lead to nuclear-wide phosphorylation of H2AX, hinting at a possible broader role of this biochemical modification in undamaged chromatin (Meyer et al., 2013). All these findings imply that the sole presence of γH2AX should not be treated as a direct proof that a discontinuity in the DNA backbone occurred and thus, γH2AX signals should be treated with great caution.

While undeniably the most popular, γH2AX is only one of many DDR-related markers that can be used for assessing the level of DNA damage. Within the ever-growing list of known DDR players RAD51, 53BP1, pRPA and pKAP1 gained the most interest in recent years (Fernandez-Capetillo et al., 2002; Mochan et al., 2004; Zellweger et al., 2015; Hill et al., 2018). However, the usability of these biomarkers is limited—first, similarly to γH2AX, detection of DDR-related proteins does not unambiguously inform about the presence of DNA strand breaks (Soutoglou & Misteli, 2008; Iijima et al., 2018) and second, there are important technical shortcomings of all IF-based assays that should be taken into consideration (Manning et al., 2012; Schnell et al., 2012). The most impactful is the non-specific binding of primary and/or secondary antibodies that results in background signal which ruins both the specificity and sensitivity of the assays. As a result, true signals are difficult to detect and often irreversibly lost. Also, due to poor signal-to-noise ratio and the variety of patterns formed by different markers (fluorescent foci of different sizes, with irregular shapes and blurred edges, pan-nuclear staining) objective quantification of DNA damage level remains challenging.

## Comet assay

Although sometimes thought of as old-fashioned, the comet assay is currently one of the most popular methods used for DNA damage level measurements, especially in the genotoxicity testing field. The concept of the method, which combines single-cell electrophoresis with fluorescent microscopy is quite simple - negatively charged DNA migrates towards the anode when DNA breaks are present. The more DNA breaks there are, the farther the migration, which is then seen in the form of so-called ‘comet tails’ (Ostling and Johanson, 1984; Singh et al., 1988). While the undisputed advantages of the method are a relatively easy and cheap protocol and the lack of reliance on DDR pathways, several shortcomings exist that render it rather unattractive for the DDR drug discovery field. First of all, despite more than 30 years of usage, it is not entirely understood how the comets are formed and what is the meaning of “hedgehog” comets, in which most of the DNA is found within the comet tail (Lorenzo et al., 2013). Furthermore, there seems to be a confusion regarding the capabilities of different variants of the method, with some researchers mistakenly referring to alkaline and neutral microgel electrophoresis as being able to specifically measure SSBs or DSBs (Collins et al., 2008). Another aspect is the variability in the readouts from different experiments, which makes it difficult to derive solid conclusions. While the inter-laboratory variability might be due to a widespread use of the method and a lack of standards, intra-laboratory variability issues point at a weakness of the protocol as being sensitive to human error. It was reported that the details of the applied protocol may affect spontaneous occurrence of DNA breaks and then the quantitative characteristics of the analysed tails (Azqueta et al., 2019). Finally, the sensitivity of the method might be too low to detect subtle effects caused by the DDR inhibitors, as it is approximated that at least 100 DNA breaks per cell are needed to produce meaningful results (Collins et al., 2008). Despite the aforementioned limitations, the comet assay and similar techniques, such as pulsed-field gel electrophoresis (Kawashima et al., 2017) remain useful techniques for DNA repair studies.

## DNA breaks profiling

While it was somewhat natural for the next-generation sequencing (NGS) methods to leave a footprint on the DNA damage detection field, in recent years we have seen an unexpected increase in the number of available methods for genome wide DNA breaks profiling at nuclear resolution. The breakthrough came with the BLESS technique introduced by the Crosetto group (Crosetto et al. 2013), in which DSBs in fixed and isolated nuclei are enzymatically processed *in situ*, which results in the attachment of a biotinylated linker that contains a barcode sequence. Genomic DNA is then extracted, captured with streptavidin beads and finally PCR amplified and sequenced. Improvements to the BLESS protocol have led to the introduction of BLISS, i-BLESS and sBLISS variants as well as other similar techniques, such as END-seq, DSB-capture, CNCC-seq and qDSB-Seq (Tsai et al., 2015; Canela et al., 2016; Lensing et al., 2016; Yan et al., 2017; Zhu et al., 2019; Bouwman et al., 2020). Among other improvements, these methods allowed a reduction in the amount of input material and in the occurrence of false positive readouts, enhanced sensitivity and increased the scalability of the assays. Importantly, variants of these approaches which focus on SSBs detection also exist (Sriramachandran et al., 2020). The capability of detailed mapping of DNA breaks and correlating them with specific sequences in the genome are the strengths of the profiling methods. Such high-level analysis may be a promising approach in early-stage target validation and high-level, high-throughput genetic screening.

Despite those very important advances, several limitations of the techniques remain, which makes it difficult to apply them on a regular basis in DNA repair related studies and drug development. There are important downsides from a technological perspective - artificial DSBs might be introduced during the fixation procedures, the minimal number of cells required for these assays to produce meaningful results is still high and they may not be suitable for all types of cells (Canela et al., 2016; Lensing et al., 2016). Biology-wise, as these methods inherently do not allow to obtain data from a single cell, information about the heterogeneity within a population is irreversibly lost. From a practical perspective, the obvious drawbacks are laborious and time-consuming protocols (e.g., 2 weeks for completion of sBLISS) and the requirement of advanced and complex computational methods and tools for data analysis (Zhu et al., 2019), which translate into high costs, low efficiency and low throughput. However, the most important problem is related to the fact that these techniques are not directly quantifiable, i.e., the results are usually presented as relative frequencies rather than numbers of DSBs per cell which precludes comparison between samples. Although the recently introduced qDSB-Seq method provides a means for overcoming this drawback by using spike-in DSBs induced by a restriction enzyme, it remains to be seen whether its experimental and computational complexity will allow for a widespread implementation of this approach.

## Direct and sensitive microscopy detection of DNA breaks

The critical pitfalls of existing IF-based methods for DNA damage detection are the lack of specificity, poor sensitivity and their reliance upon active DDR mechanisms. We have recently introduced STRIDE (Kordon et al., 2020), a fluorescence-based method which, as we have shown, overcomes these limitations, but at the same time allows to retain the flexibility of IF assays and fully exploit the capabilities of modern fluorescence confocal microscopy. STRIDE enables direct *in situ* labelling and detection of single- or double-strand DNA breaks in any type of biological material. The method is based on enzymatic modification of free DNA ends by incorporation of modified nucleotides with subsequent PLA-based detection of these modifications. Two basic variants of the technology exist (sSTRIDE and dSTRIDE), which in most cases enable distinction between SSBs and DSBs by utilization of different enzymes (Polymerase I, PolI or Terminal Deoxynucleotidyl Transferase, TdT). STRIDE in various ways can be considered superior to the existing methods of DNA damage detection. First, it is direct and sensitive—features that were not thought to be possibly combined in a single assay before. The lower limit of detection of STRIDE is one (individual) single- or double-strand DNA break, which ensures an unprecedented sensitivity level of the assay. Second, the undeniable strength of the method is its specificity, which is achieved by incorporation of exogenous molecules (modified nucleotides) to the cells, minimizing non-specific binding of antibodies and further ensured by the principles of Proximity Ligation Assay (Fredriksson et al., 2002; Söderberg et al., 2006). The latter also contributes to the enhanced sensitivity, as it permits strong signal amplification even at sites of individual DNA breaks. It is noteworthy to mention that STRIDE informs about events beyond the cell nucleus, as it can be used to quantify the level of cytosolic DNA fragments. The qualities of STRIDE are evident in microscopy images of samples in which DNA breaks were detected using this technique - DNA breaks are visible as distinct, fluorescence foci with very high signal-to-noise ratio on a near zero background. The nature of the resulting signal also has a direct impact on the quality and objectivity of data quantification, as separation of true signal from the background is easily achievable and can be automated. Importantly, STRIDE can be performed on multi-well plates and thus easily adapted for high-throughput screening. While confocal microscopy imaging is preferable to allow precise quantification of individual STRIDE foci, widefield fluorescence microscopy can be used to measure the overall intensity of the signals.

As the *in situ* modification of DNA ends is a step that is somewhat similar in STRIDE and DSBs profiling methods, these approaches also share some of the shortcomings. It is currently unknown what is the percentage of DNA breaks that are not enzymatically modified after cell fixation. The reasons for the lack of incorporation of modified nucleotides are at least twofold - these are chromatin compaction and overall steric hindrance around the DNA break and the presence of damaged, so called ‘dirty’ DNA ends, i.e., ends lacking a necessary 3′OH moiety. These problems are mitigated in the STRIDE protocol in sample preparation steps which allow to increase chromatin accessibility and convert damaged DNA ends into substrates appropriate for the enzymes used. The number of DNA breaks detected by STRIDE might be further underestimated when the lesions are clustered, leaving little space for the antibodies to bind or resulting in fluorescence foci located at a distance that is below the resolving power of standard wide-field or fluorescence confocal microscopy. The latter problem can be partially overcome by using super-resolution microscopy, but that would come with a trade-off in throughput and scalability. Also, it is important to note that while dSTRIDE is specific to DSBs, sSTRIDE is potentially capable of producing signals at 5′overhangs of a DSB, apart from DNA nicks and ssDNA gaps. Appropriate modification of the protocol, i.e., blunting of DNA ends before PolI enzymatic reaction can be applied to resolve this issue.

## Discussion

General expectations of drug developers regarding PD biomarkers are usually quite similar. First, they should be as proximal as possible, i.e., informing about a process that is directly or very closely related to the target. Second, it is expected for a PD biomarker to immediately report on the effect of target modulation. Finally, there are technical requirements related to specificity, sensitivity and reproducibility of a particular assay. If a PD biomarker meets at least some of these criteria, the drug development process can be facilitated in many ways. In early-stage discovery it allows to speed up drug candidate screening, pick the most promising leads and often helps to discover the mechanism of action of the compounds. Some of the tools may also be found very useful in characterisation of the targets’ biology what can be applied at a very early stage of target validation, drug discovery and design. Yet the real value of any biomarker is verified when moving into *in vivo* pre-clinical and clinical studies and very often this test ends in a failure. The expectation for a robust PD biomarker to be able to accompany each stage of drug development is thus the most important and at the same time the most difficult to meet.

**FIGURE 1:**
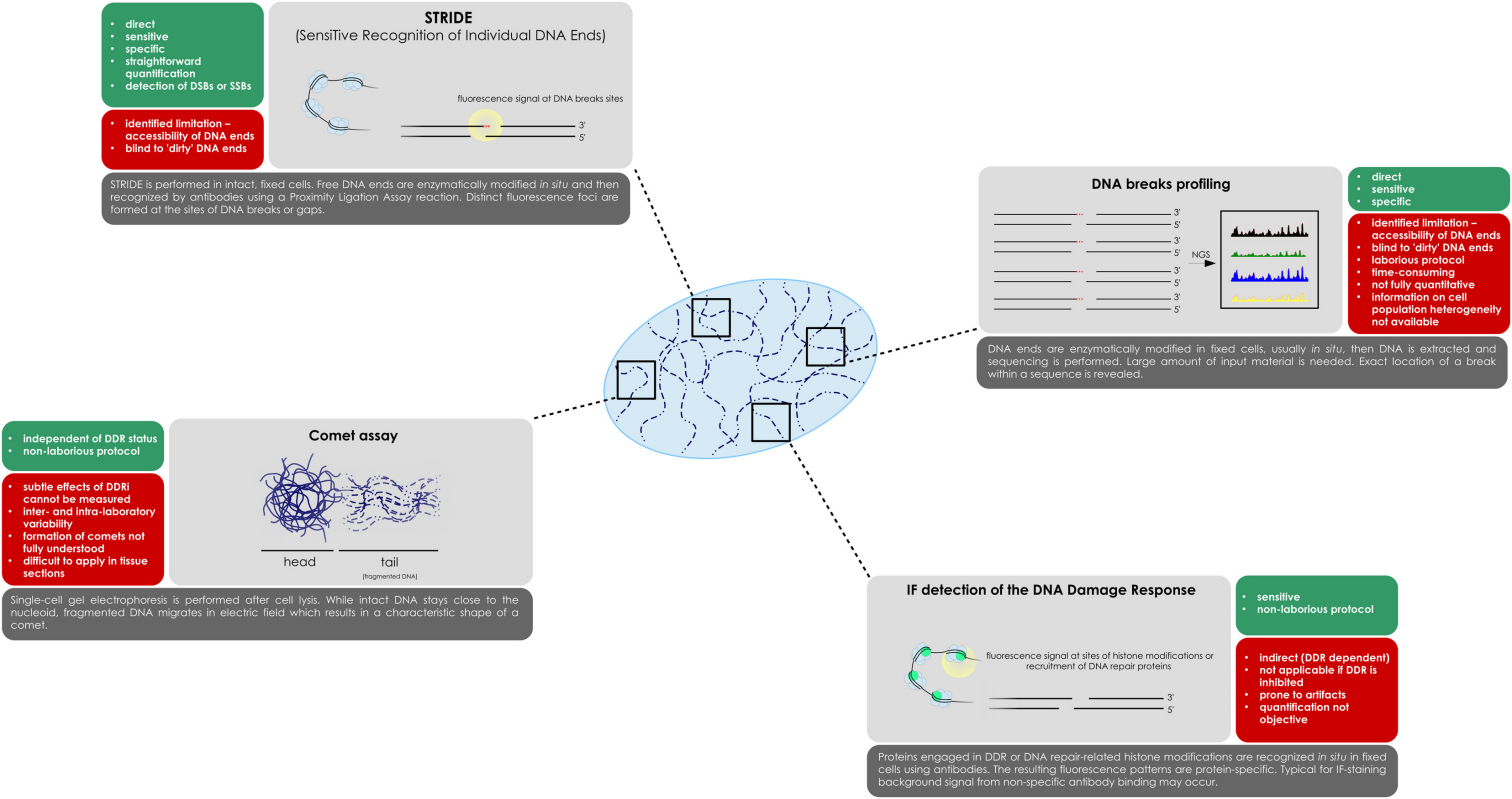
Methods for DNA breaks detection, as presented in the paper. While STRIDE, DNA breaks profiling and the comet assay are DDR-independent, IF detection of repair proteins and histone modifications relies on active DNA surveillance mechanisms. A schematic representation of each of the method is presented with a list of most important features.

The methods used for DNA damage level measurements discussed here (Figure 1) were rarely used in clinical trials in the DDR inhibitors context so far (Cleary et al., 2020; Huang and Zhou, 2021). One of the reasons behind the slow adoption of these assays is that their drawbacks, e.g., the non-specific staining in IF-based detection of repair proteins, are further exacerbated when moving from relatively easy to handle 2D cell models into more complex human material, such as FFPE tissue sections. Furthermore, it should be stressed out that inhibition of some of the DDR proteins (e.g., ATM, ATR, DNA-PK) often results in a substantiable reduction or even complete loss of downstream signalling, which precludes the use of methods based on detection of the DNA repair processes. Comet assay and DNA breaks profiling methods, on the other hand, require isolated cells, which limits their use to blood cells or implies dissociation of solid tissues, further increasing the risk of producing artifacts (Bouwman et al., 2020; Cardoso et al., 2022).

Direct and sensitive *in situ* detection of DNA breaks provided by STRIDE overcomes most of the weaknesses of the existing methods (Table 1). Finally, researchers are equipped with a comprehensive toolset that enables to measure not only DSBs but also the increasingly important ssDNA gaps. There are currently numerous studies underway in which STRIDE is implemented to report on the efficiency of different DDR inhibitors. To date, the technique has been used to study events related to USP1 or PARP7 inhibition and formation of chromosome bridges (Gozgit et al., 2021; Rodriguez-Muñoz et al., 2021; Simoneau et al., 2022). Preliminary validation of the assay in FFPE tissue sections and the already proven applicability of the PLA methodology in human tissues (Hegazy et al., 2020; Lindskog et al., 2020) give hope that this technology may be successfully used in further stages of the drug development process. However, a full validation and a proof-of-concept clinical study are needed to provide solid proof that STRIDE indeed has the potential to transform the landscape of DDR functional biomarkers.

**TABLE 1:** Methods for DNA breaks detection.

	Sensitivity	Minimal no. of DNA breaks	Direct detection of DNA breaks	*in situ* detection	Single-cell information	Distinction between types of DNA breaks	Combination with other biomarkers
**IF/IHC detection of DDR**	high	1-50^*^	no	yes	yes	no	yes
**Comet assay**	medium	100^**^	no	no	no	no	no
**DNA breaks profiling**	very high	1^***^	yes	yes	no	yes	no
**STRIDE**	very high	1	yes	yes	yes	yes	yes

* Detection of a single DSB, was only demonstrated for γH2AX (Soutoglou et al., 2007). For methods detecting other DDR, events, such as accumulation of RAD51 or RPA proteins the minimal no. of DNA breaks is not known precisely.

** This value was approximated based on the ionizing radiation dose that is needed to produce a detectable signal (Collins et al., 2008).

*** Although a single DNA break can be enzymatically modified *in situ* in early steps of the procedures, data cannot be generated from an individual break in a single cell.
